# Outcomes and time trends of acute respiratory distress syndrome patients with and without liver cirrhosis: an observational cohort

**DOI:** 10.1186/s13613-023-01190-1

**Published:** 2023-09-29

**Authors:** Joris Pensier, Audrey De Jong, Clément Monet, Yassir Aarab, Clément Le Bihan, Mathieu Capdevila, Inès Lakbar, Lucas Stock, Fouad Belafia, Gerald Chanques, Nicolas Molinari, Samir Jaber

**Affiliations:** 1 Anesthesiology and Intensive Care; Anesthesia and Critical Care Department B, Saint Eloi Teaching Hospital, PhyMedExp, University of Montpellier, INSERM U1046, 1, 80 Avenue Augustin Fliche, Montpellier Cedex 5, Montpellier, France; 2 Centre Hospitalier Universitaire Montpellier, 34295 Montpellier, France; 3 CEReSS, Health Service Research and Quality of Life Centre, School of Medicine, Aix-Marseille University, La Timone, Marseille, France; 4 Medical Information, IMAG; CNRS, Univ Montpellier, Centre Hospitalier Regional Universitaire de Montpellier, Montpellier, France; 5 Institut Desbrest de Santé Publique (IDESP), INSERM-Université de Montpellier. Département d’informatique Médicale, CHRU Montpellier, Montpellier, France; 6 Samir JABER, Département d’Anesthésie Réanimation B (DAR B), 80 Avenue Augustin Fliche, 34295 Montpellier, France

**Keywords:** Intensive care unit, Acute respiratory distress syndrome, Ventilation, Liver failure, Cirrhosis

## Abstract

**Background:**

In studies prior to lung-protective ventilation, liver cirrhosis in acute respiratory distress syndrome (ARDS) was associated with high mortality rates. Since patients with cirrhosis have been excluded from many trials on ARDS, their outcome when treated with lung-protective ventilation is unclear. The objectives were to assess whether cirrhosis is associated with mortality in ARDS and trends over time in mortality and severity.

**Methods:**

We conducted a retrospective analysis of a prospective observational cohort conducted in a 20-bed tertiary ICU from October 2003 to December 2021. All consecutive adult critically ill patients with ARDS were included. ARDS was defined by the Berlin criteria. The primary outcome was 90 day mortality, assessed with Kaplan–Meier curves and multivariate Cox analysis. Time trends were assessed on 90 day mortality, Sequential Organ-Function Assessment score (SOFA) and non-hepatic SOFA. Ventilation settings were compared between patients with and without cirrhosis.

**Results:**

Of the 7155 patients screened, 863 had a diagnosis of ARDS. Among these ARDS patients, 157(18%) had cirrhosis. The overall 90 day mortality was of 43% (378/863), 57% (90/157) in patients with cirrhosis and 41% (288/706) in patients without cirrhosis (*p* < 0.001). On survival curves, cirrhosis was associated with 90 day mortality (*p* < 0.001). Cirrhosis was independently associated with 90 day mortality in multivariate analysis (hazard ratio = 1.56, 95% confidence interval 1.20–2.02). There was no change in mortality over time in ARDS patients with and without cirrhosis. SOFA (*p* = 0.04) and non-hepatic SOFA (*p* = 0.02) increased over time in ARDS patients without cirrhosis, and remained stable in ARDS patients with cirrhosis. Tidal volume, positive end-expiratory pressure, plateau pressure and driving pressure were not different between ARDS patients with and without cirrhosis.

**Conclusions:**

Although ARDS management improved over the last decades, the 90 day mortality remained high and stable over time for both ARDS patients with (57%) and without cirrhosis (41%). Nevertheless, the severity of patients without cirrhosis has increased over time, while the severity of patients with cirrhosis has remained stable.

**Graphical Abstract:**

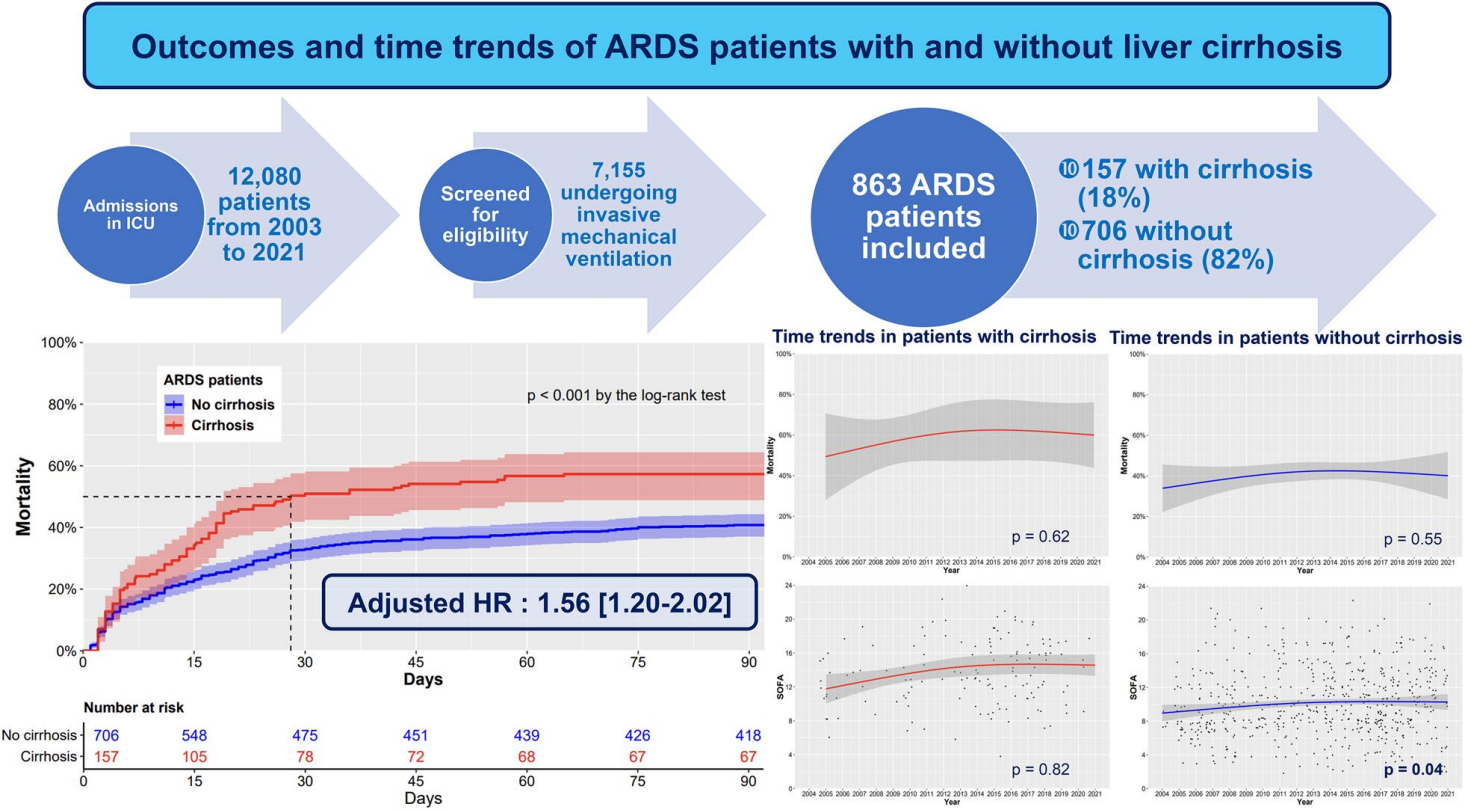

**Supplementary Information:**

The online version contains supplementary material available at 10.1186/s13613-023-01190-1.

## Background

The acute respiratory distress syndrome (ARDS) is a serious condition with a high mortality, ranging from 35 to 50% [1–3]. In studies performed before 2000, liver cirrhosis in ARDS patients was associated with an even higher mortality, ranging from 67 to 96% [4–6]. Since then, research has provided new insights on ARDS that have resulted in major clinical implications [7, 8]. Randomized controlled trials have strengthened their methodology over time [9], and lung-protective ventilation has emerged as a standard of care [10, 11]. A decrease of mortality in ARDS since the 1990’s has been found [12–14]. However, patients with cirrhosis have been excluded from 9 out of 15 randomized controlled trials on ARDS performed between 2005 and 2015 [1, 15, 16], and recent trials continue to list cirrhosis as an exclusion criterion [17, 18].

At the same time, the proportion of critically ill patients with comorbidities in overall ARDS patients is increasing. Half of the ARDS patients have a major comorbidity [15]. In a study [15], cirrhosis was present in 7.2% of ARDS patients and led to twice as many treatment-limitation decisions in Intensive Care Unit (ICU) than patients without comorbidity. However, cirrhosis was not associated with 28 day mortality in a multivariate analysis in this study. In a secondary analysis of ACURASYS and PROSEVA by Dizier and al., early hepatic dysfunction was associated with mortality, but no distinction was made between acute, chronic and acute-on-chronic liver failure [19].

In this setting, it is unclear whether the prognosis of ARDS patients with cirrhosis has improved along with the breakthroughs done in the management of the overall population of ARDS patients [15], or if cirrhosis remains associated with worse outcomes in ARDS.

Our primary objective was to assess whether cirrhosis is associated with the 90 day mortality in ARDS patients. Our secondary objectives were to assess changes over time in mortality and severity of ARDS patients with and without cirrhosis, and to compare ventilation settings in ARDS patients with and without cirrhosis. Our hypothesis was that cirrhosis remains associated with mortality in ARDS with no decrease over the study period, despite ventilation settings similar in patients with and without cirrhosis.

## Material and methods

### Study design

We conducted a retrospective analysis of prospectively collected data of all ARDS adult patients consecutively admitted to a 20-bed mixed medical-surgical adult ICU in a university teaching hospital between October 2003 and December 2021. We obtained approval from the Montpellier University Hospital ethics committee for the Forever project on May 29, 2019 (Comité Local d’Ethique Recherche, agreement number: 2019_IRB-MTP_05-25). The study has been performed in accordance with the Helsinki declaration of 1975.

### Data collection

Patients were identified through our computerized database. ARDS was identified based on the Berlin criteria consensus definition[20]: (1) timing: onset within 1 week of a known clinical insult or new or worsening respiratory symptoms; (2) chest imaging: bilateral opacities, not fully explained by effusions, lobar/lung collapse or nodules; (3) origin of edema: respiratory failure not fully explained by cardiac failure or fluid overload; (4) PaO_2_/FiO_2_ ratio (PaO_2_/FiO_2_) ≤ 300 mmHg with positive end-expiratory pressure (PEEP) or continuous positive airway pressure ≥ 5 cm H2O. ARDS diagnosis was retrospectively reviewed by two physicians (JP and ADJ). As performed in previous studies [21–23], the diagnosis of cirrhosis was based on previous histology findings when available or on various associations of clinical, biological, endoscopic, and/or ultrasonographic or imaging findings, including cutaneous manifestations such as jaundice or skin telangiectasias, evidence of portal hypertension such as variceal bleeding, ascites, hepatic encephalopathy, and biological results of liver failure. All consecutive adult patients with ARDS undergoing invasive mechanical ventilation hospitalized during the study period in ICU were included in the study. Only the first admission of each patient with ARDS was retained for analysis. Patient data were recorded by the ICU’s physicians and included demographics, anthropometrics, clinical and biological data on ICU admission, as well as the Sequential Organ Failure Assessment (SOFA score) [24], the Simplified Acute Physiology Score II (SAPS II) and comorbidities. The SOFA score was split in non-hepatic SOFA and hepatic SOFA. Ventilator-associated pneumonia was diagnosed as previously published [25]. All ventilation data (plateau pressure, tidal volume (Vt), PEEP, driving pressure (plateau pressure–PEEP), respiration rate) were recorded at day 1 of the ARDS onset. Compliance of the respiratory system (Crs) was calculated using the formula Vt/(plateau pressure–PEEP) [2]. ARDS severity was assessed according to the Berlin definition (severe ARDS: PaO_2_/FiO_2_ < 100 mmHg, moderate: PaO_2_/FiO_2_ [100–200 mmHg], mild: PaO_2_/FiO_2_ > 200 mmHg) [20], based on the worse PaO_2_/FiO_2_ at day 1 of ARDS onset. Child–Pugh score was assessed on the day of ICU admission and calculated as previously described [22]. The Model for End-Stage Liver Disease (MELD) score was calculated based on the laboratory results obtained on admission in the ICU [26]. All included patients were ventilated with a lung-protective mechanical ventilation protocol as defined in the literature: objective of low Vt of 6 ml/kg of predicted body weight (PBW), limited plateau pressure [2]. Ventilatory parameters were set to avoid intrinsic PEEP.

### Outcomes

The primary outcome was the mortality at 90 days (90 day mortality), obtained using hospital electronic patient records. The secondary outcomes were the evolution of 90 day mortality over the study period, the evolution of the SOFA score and the non-hepatic SOFA score over the study period, and ventilatory parameters at day one of ARDS onset.

### Statistical analysis

First, a descriptive analysis was performed overall in cirrhotic and non-cirrhotic patients. Quantitative variables were expressed as mean [standard deviation (SD)] or median (interquartile range, 25–75%) and compared using the Student-t test or Wilcoxon test as appropriate (Gaussian or non-Gaussian variables) [27]. Qualitative variables were expressed as numbers (%) and compared using the uncorrected Chi-square test or Fisher test as appropriate [27].

Survival curves were set up until day 90 using the Kaplan–Meier method and compared with the log-rank test [28]. Then we used a univariate Cox-proportional hazard model to determine whether cirrhosis was associated with 90 day mortality in an unadjusted analysis [28]. Then we used a multivariate Cox-proportional hazard model to determine whether cirrhosis was independently associated with mortality after adjustment for age, non-hepatic SOFA and the PaO2/FiO2 at day one of the ARDS onset [2]. Collinearity between variables was tested [9]. No imputation of missing data was made.

Then, we performed an exploratory analysis using a logistic regression. We performed a univariate analysis to assess the variables associated with 90 day mortality in ARDS patients. For both (Cox-proportional and logistic model), variables with a *p*-value < 0.20 in the univariate analysis were selected and a stepwise procedure was used to select the final multivariate model, according to their Akaike Information Criteria (AIC) [9].

Then, we performed an exploratory analysis excluding the patients included in randomized controlled trials. Survival curves were set up until day 90 using the Kaplan–Meier method and compared with the log-rank test [28]. Then we used a univariate Cox-proportional hazard model to determine whether cirrhosis was associated with 90 day mortality in an unadjusted analysis [28]. Then we used a multivariate Cox-proportional hazard model to determine whether cirrhosis was independently associated with mortality after adjustment for age, non-hepatic SOFA and the PaO2/FiO2 at day one of the ARDS onset [2].

Time trends in day-90 mortality in ARDS patients with cirrhosis and without cirrhosis were evaluated with the Cochran–Armitage test [15]. Time trends in SOFA score and non-hepatic SOFA score were evaluated with the Mann–Kendall test [29] (to assess monotonic trends over time) and the Pettitt’s test [30] (to detect change-points over time).

Tests were two-sided and *p* < 0.05 were considered significant. We used R (version 4.2.0) to perform all statistical analyses. The study was reported according to the STrengthening the Reporting of OBservational studies in Epidemiology (STROBE) reporting guideline statement [31].

## Results

### Patients

**Fig. 1:**
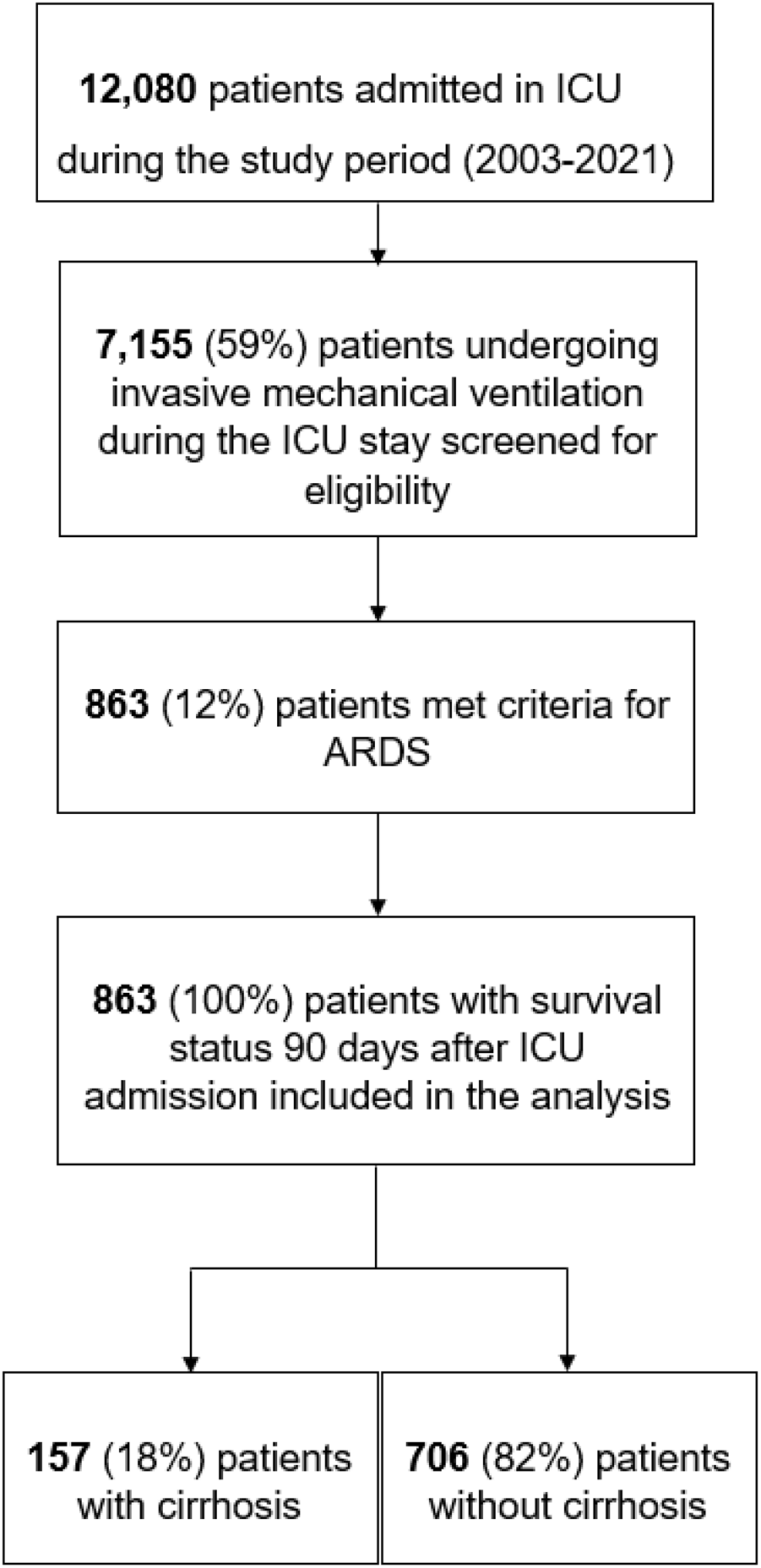
Flowchart of the study

During the study period (2003–2021), 12,080 patients were admitted in ICU, and 7155 (59%) patients underwent mechanical ventilation. Among those patients, 863 (12%) met criteria for ARDS. Of these patients, 863 (100%) had an available survival status 90 days after ICU admission and were included in the analysis. Among these 863 patients, 157 (18%) were ARDS patients with cirrhosis and 706 (82%) were ARDS patients without cirrhosis. Figure 1 presents the flowchart of the study. One hundred and forty-two patients (16%) had been included in prospective randomized trials: 12 in the EXPRESS study [32], 26 in the ACURASYS study [33], 12 in the PROSEVA study [34], 42 in the BIRDS study (NCT01862016), and 49 in the LIVE study [18] and one in the HIGH study [35]. The baseline characteristics for ARDS patients with and without cirrhosis are presented in Table 1 and Additional file 1: Table S1. The management and the outcomes of ARDS patients are presented in Table 2.

**Table 1:** Baseline characteristics in 863 ARDS patients with and without cirrhosis

Characteristics	Total (%) (*n* = 863)	Patients with cirrhosis (*n* = 157)	Patients without cirrhosis *(n* = 706)	*p* value
Age (years)	60 (± 15)	56 (± 11)	62 (± 15)	< 0.001
Sex				0.10
Male	597 (69%)	119 (76%)	478 (68%)	
Female	266 (31%)	38 (24%)	228 (32%)	
BMI (kg/m^2^), mean (± SD)	25.9 (± 5.9)	25.2 (± 5.4)	26.0 (± 5.8)	0.24
SAPS II, mean (± SD)	52 (± 18)	53 (± 18)	52 (± 18)	0.62
SOFA, mean (± SD)^a^	11 (± 4)	13 (± 4)	10 (± 4)	< 0.001
Non-hepatic SOFA, mean (± SD)^a^	10 (± 4)	11 (± 3)	9 (± 4)	< 0.001
Hepatic SOFA, mean (± SD)^a^	1 (± 1)	2 (± 1)	1 (± 1)	< 0.001
Cause of ARDS				0.01
Medical	480 (56%)	102 (65%)	378 (54%)	
Postoperative	383 (44%)	55 (35%)	328 (46%)	
Origin of ARDS				0.41
Pulmonary	479 (55%)	82 (52%)	397 (56%)	
Pneumonia	204 (24%)	36 (23%)	168 (24%)	0.80
Extrapulmonary	384 (45%)	75 (48%)	309 (44%)	
Severity of ARDS				0.04
Mild	315 (36%)	45 (28%)	270 (39%)	
Moderate	425 (49%)	83 (53%)	342 (48%)	
Severe	123 (14%)	29 (19%)	94 (13%)	
PaO_2_/FiO_2_ (mmHg), mean (± SD)	180 (± 95)	168 (± 82)	183 (± 97)	0.58
PaCO_2_ (mmHg), mean (± SD)	40 (± 4)	40 (± 4)	40 (± 4)	0.84
Lactate, mean (± SD)	2.1 (± 1)	3.2 (± 2)	1.9 (± 1)	0.01
pH, mean (± SD)	7.33 (± 0.13)	7.30 (± 0.16)	7.33 (± 0.12)	0.15
Bicarbonate (mmol/L), mean (± SD)	24 (± 13)	22 (± 5)	24 (± 14)	0.17

SD: standard deviation, BMI: body mass index, SAPS II: Simplified Acute Physiology Score II, SOFA: Sequential Organ Failure Assessment, ARDS: acute respiratory distress syndrome, PaO_2_/FiO_2_: PaO_2_/FiO_2_ ratio

^a^Forty (5%) missing values

**Table 2:** ARDS management and outcomes in 863 ARDS patients with and without cirrhosis

Characteristics	Total (%) (*n* = 863)	Patients with cirrhosis (*n* = 157)	Patients without cirrhosis (*n* = 706)	*p* value
Prone positioning	317 (37%)	47 (30%)	270 (38%)	0.06
NMBA	485 (56%)	78 (50%)	407 (58%)	0.08
Inhaled nitric oxide	81 (9%)	7 (4%)	74 (10%)	0.03
Corticosteroids	354 (41%)	129 (83%)	225 (32%)	< 0.001
ECMO	18 (2%)	2 (1%)	16 (2%)	0.43
Ventilator-associated pneumonia	156 (18%)	32 (20%)	124 (18%)	0.87
MV-free days at 28 day, mean (± SD)	21 (± 7)	22 (± 6)	21 (± 7)	0.18
ICU length of stay, mean (± SD)	14 (± 13)	15 (± 12)	14 (± 13)	0.07
Hospital length of stay, mean (± SD)	23 (± 24)	19 (± 23)	24 (± 24)	0.01
7 day mortality	146 (17%)	37 (24%)	109 (15%)	0.01
28 day mortality	308 (36%)	78 (50%)	230 (33%)	< 0.001
90 day mortality	378 (44%)	90 (57%)	288 (41%)	< 0.001

ARDS: acute respiratory distress syndrome, SD: standard deviation, NMBA: neuromuscular blocking agent, ECMO: extra-corporeal membrane oxygenation, MV: mechanical ventilation, ICU: intensive care unit

Among the 157 ARDS patients with cirrhosis, 19 patients (12%) were classified Child–Pugh A, 48 patients (31%) were classified Child–Pugh B, and 90 patients (57%) were classified Child–Pugh C. The main cause of cirrhosis was alcohol in 94 patients (59%), viral hepatitis in 40 patients (26%) and other in 23 patients (15%). Among patients with cirrhosis, 31 (19%) underwent liver transplantation after the diagnosis of ARDS. Specific characteristics of ARDS patients with cirrhosis are presented in Additional file 1: Table S2. Additional file 1: Table S3 summarizes missing values for studied outcomes.

### Primary outcome: 90 day mortality

**Fig. 2:**
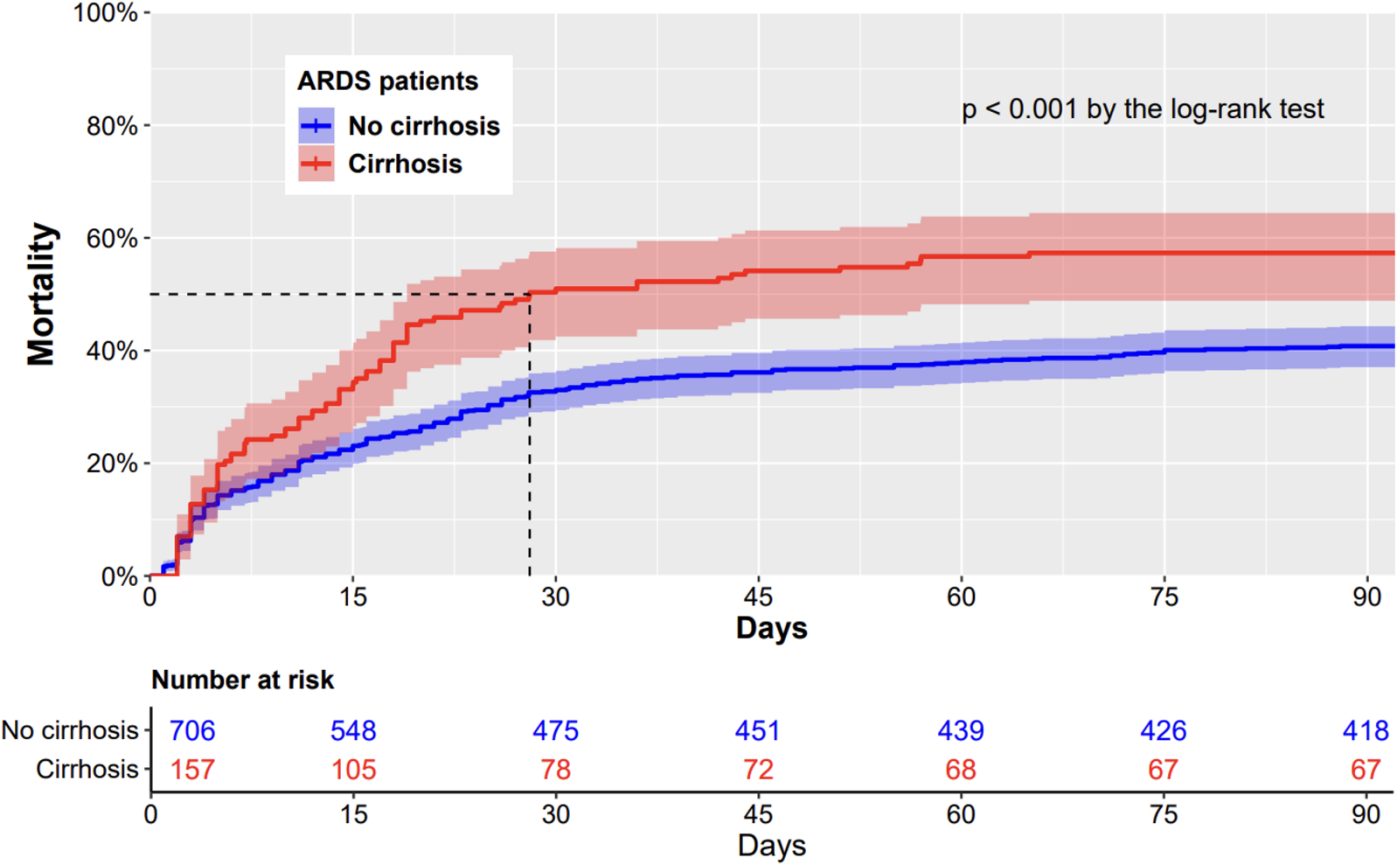
Cumulative 90 day mortality in 706 ARDS patients without cirrhosis and 157 ARDS patients with cirrhosis. *p*-value < 0.001 by the log-rank test

90 day mortality was of 43% overall (378/863). The 90 day mortality was of 57% (90/157) in the ARDS patients with cirrhosis and of 41% (288/706) in the ARDS patients without cirrhosis (*p* < 0.001). Kaplan–Meier survival curves were set up until 90 days after admission in the ICU (Fig. 2). In survival analysis, the 90 day mortality was significantly higher in ARDS patients with cirrhosis, compared to ARDS patients without cirrhosis (*p* < 0.001 by the log-rank test). In the univariate Cox-proportional hazard model, cirrhosis was significantly associated with the 90-mortality (unadjusted hazard ratio (HR) = 1.63, 95% confidence interval (95%CI) [1.28–2.06], *p* < 0.001).

In the multivariate Cox-proportional hazard model, cirrhosis was independently associated with mortality after adjustment for age, non-hepatic SOFA and the PaO2/FiO2 at day one of the ARDS onset (adjusted HR = 1.56, 95%CI [1.20–2.02], *p* < 0.001). No collinearity between variables was found (Additional file 1: Table S4.).

In the exploratory analysis using a logistic regression, age, non-hepatic SOFA, cause of ARDS, origin of ARDS, ARDS severity and use of neuromuscular blocking agents (NMBA) were selected in the univariate analysis (Additional file 1: Table S5.). After the stepwise procedure, the final multivariate model included cirrhosis, age, non-hepatic SOFA and ARDS severity (Additional file 1: Table S6). In this model, cirrhosis was independently associated with mortality (OR = 1.78, 95%CI [1.15–2.75], *p* < 0.01).

In the exploratory analysis excluding the 142 patients included in randomized controlled trials, Kaplan–Meier survival curves were set up until 90 days after admission in the ICU (Additional file 1: Fig. S1.). In survival analysis, the 90 day mortality was significantly higher in ARDS patients with cirrhosis, compared to ARDS patients without cirrhosis (*p* < 0.001 by the log-rank test). In the univariate Cox-proportional hazard model, cirrhosis was significantly associated with the 90-mortality (unadjusted hazard ratio (HR) = 1.61, 95% confidence interval (95%CI) [1.25–2.09], *p* < 0.001). In the multivariate Cox-proportional hazard model, cirrhosis was independently associated with mortality after adjustment for age, non-hepatic SOFA and the PaO2/FiO2 at day one of the ARDS onset (adjusted HR = 1.41, 95%CI [1.06–1.87], *p* = 0.01).

### Time trends for 90 day mortality and SOFA score

**Fig. 3:**
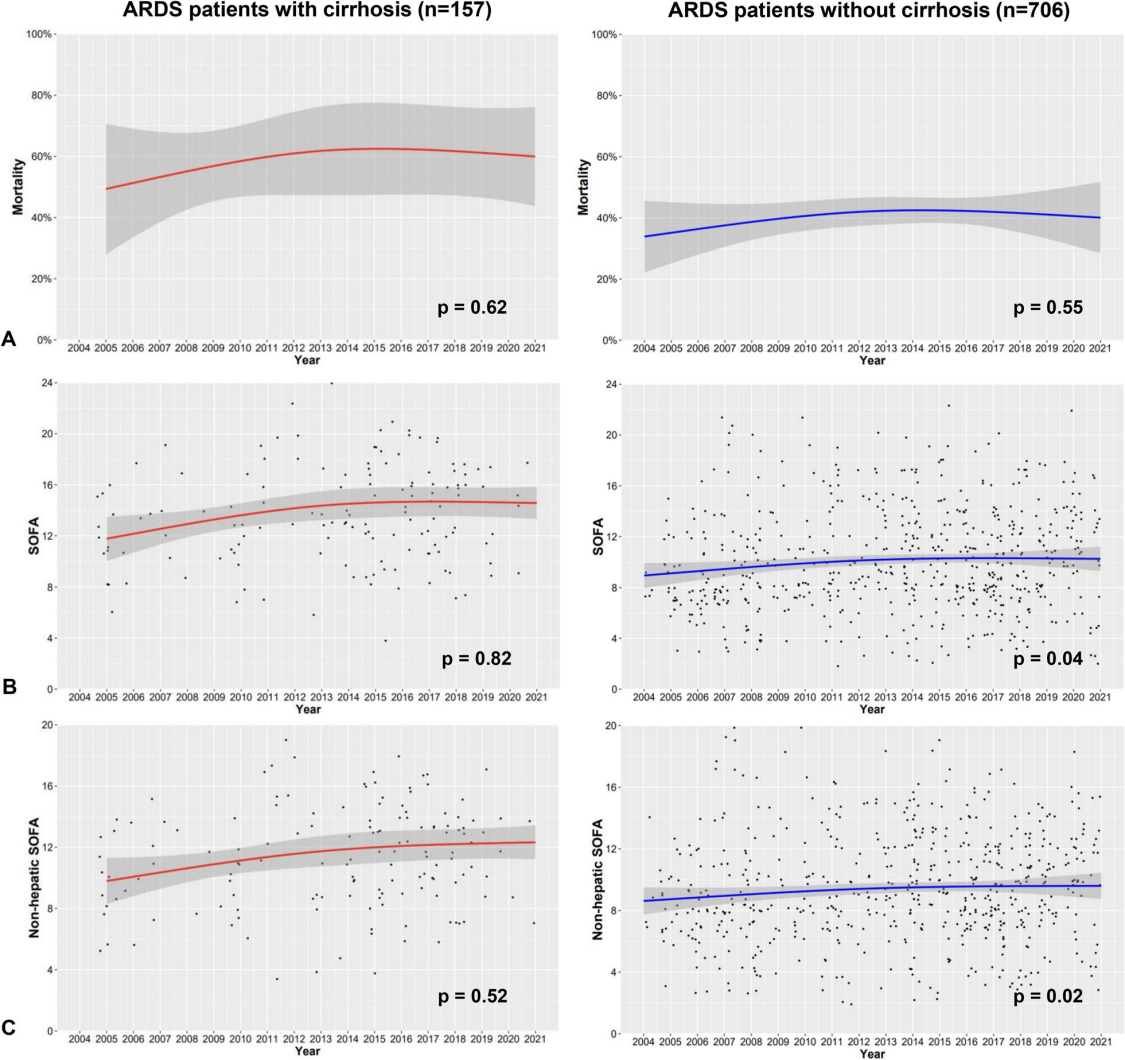
Time trends over the study period in 863 ARDS patients with and without cirrhosis. **A** 90 day mortality. 90 day mortality (with standard error) during each study year in ARDS patients without cirrhosis (blue bars) and ARDS patients with cirrhosis (red bars). The test for trend was non-significant in the patients without cirrhosis (Cochran–Armitage test, *p =* 0.55) and in patients with cirrhosis (Cochran–Armitage test, *p *= 0.62). **B** SOFA score on ICU admission. SOFA score during each study year in ARDS patients without cirrhosis (blue boxes) and ARDS patients with cirrhosis (red boxes). SOFA: Sequential Organ Failure Assessment. **C** Non-hepatic SOFA score on ICU admission. SOFA score during each study year in ARDS patients without cirrhosis (blue boxes) and ARDS patients with cirrhosis (red boxes). SOFA: Sequential Organ Failure Assessment

Figure 3A presents the mortality of ARDS patients each year between 2003 and 2021 for patients with cirrhosis and patients without cirrhosis. There was no significant change over time in the 90 day mortality of ARDS patients with cirrhosis (Cochran–Armitage test, *p* = 0.62). There was no significant change over time in the 90 day mortality of ARDS patients without cirrhosis (Cochran–Armitage test, *p* = 0.55).

In ARDS patients with cirrhosis, there was no significant change over time in the SOFA score (Mann–Kendall test for linear trend: *p* = 0.82, Pettit’s test for change-point: *p* = 0.36, Fig. 3B). In these patients, there was no significant change over time in the non-hepatic SOFA score (Mann–Kendall test: *p* = 0.52, Pettit’s test: *p* = 0.33, Fig. 3C).

In ARDS patients without cirrhosis, SOFA score increased significantly over time (Mann–Kendall test for linear trend: *p* = 0.04, Fig. 3B.), with no detected change-point (Pettit’s test for change-point: *p* = 0.21). In these patients, non-hepatic SOFA score increased significantly over time (Mann–Kendall test: *p* = 0.02, Fig. 3C.) with a change-point detected in 2011 (Pettit’s test: *p* = 0.01).

### Ventilatory parameters at day one of ARDS onset

**Fig. 4:**
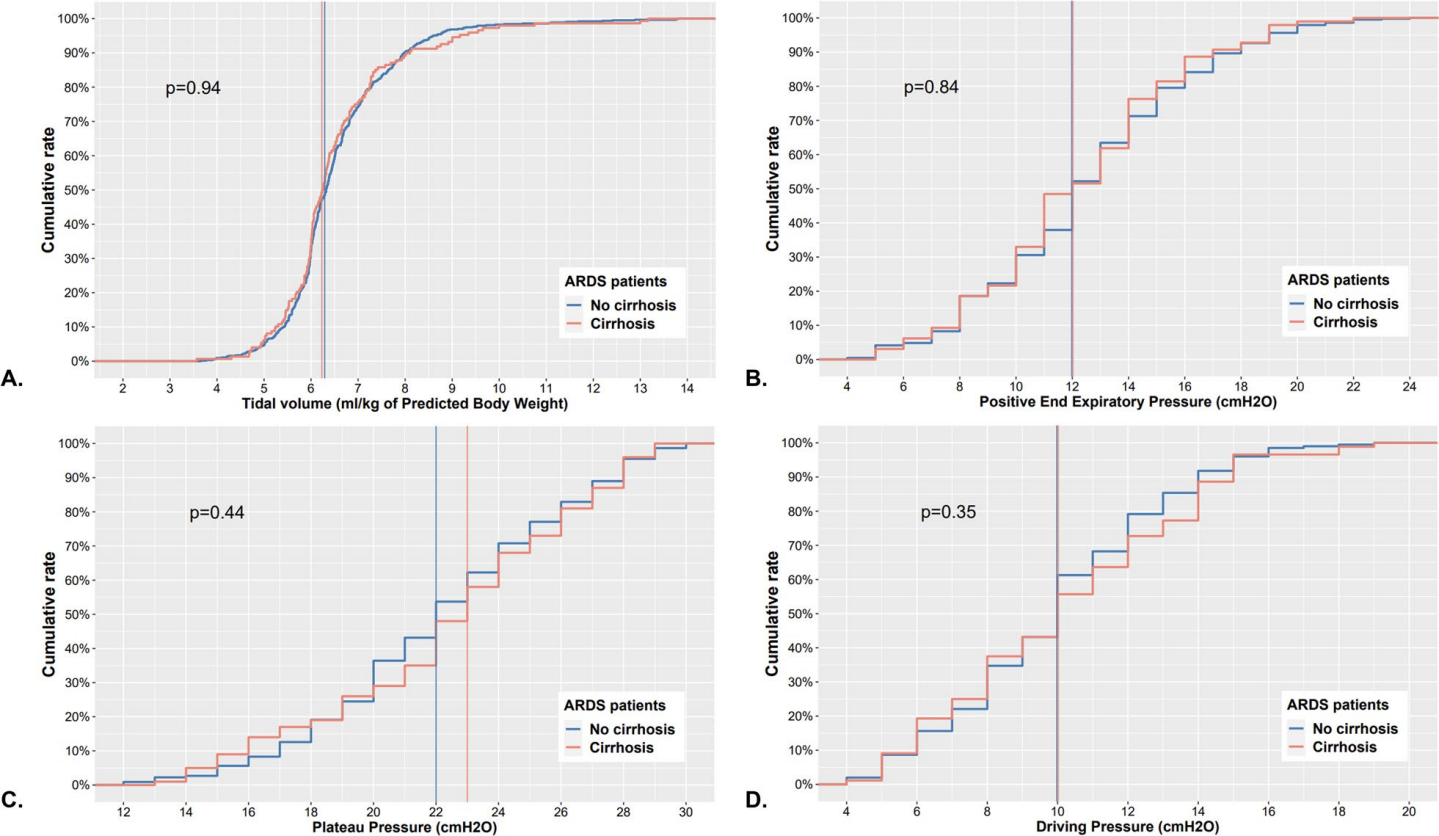
Cumulative frequency graphs for ventilatory parameters in ARDS patients with and without cirrhosis. **A** Tidal volume. **B** Positive end-expiratory pressure.** C** Plateau pressure.** D** Driving pressure

Overall, the median Vt was of 6.3 ml/kg of PBW (IQR [5.9–7.0]). There was no significant difference in the set Vt in ARDS patients with cirrhosis compared to ARDS patients without cirrhosis (*p* = 0.94, Fig. 4A.).

Overall, the median PEEP was of 12 cmH2O (IQR [10–14]). There was no significant difference in the set PEEP in ARDS patients with cirrhosis compared to ARDS patients without cirrhosis (*p* = 0.84, Fig. 4B.).

Overall, the median plateau pressure was of 22 cmH2O (IQR [19–25]). There was no significant difference in the plateau pressure in ARDS patients with cirrhosis compared to ARDS patients without cirrhosis (*p* = 0.44, Fig. 4C.).

Overall, the median driving pressure was of 10 cmH2O (IQR [8–12]). There was no significant difference in the driving pressure in ARDS patients with cirrhosis compared to ARDS patients without cirrhosis (*p* = 0.35, Fig. 4D.).

Additional file 1: Table S7 presents the ventilatory parameters set at day one of ARDS onset. The median Crs was of 41 ml/cmH2O [IQR (32–53)]. There was no significant difference in the Crs in ARDS patients with cirrhosis compared to ARDS patients without cirrhosis (*p* = 0.96, Additional file 1: Fig. S2.). There was no difference in the Respiration Rate in ARDS patients with cirrhosis compared to ARDS patients without cirrhosis (*p* = 0.40, Additional file 1: Fig. S3.).

Figures S4 to S9 present the trends over time for ventilatory parameters in ARDS patients with and without cirrhosis. In ARDS patients with cirrhosis, the tidal volume did not significantly change over time (Additional file 1: Fig. S4, *p* = 0.06), the PEEP increased over time (Additional file 1: Fig. S5, *p* < 0.01), the plateau pressure did not significantly change over time (Additional file 1: Fig. S6, p = 0.25), while the driving pressure decreased over time (Additional file 1: Fig. S7, *p* < 0.001) and the Crs increased over time (Additional file 1: Fig. S8, *p* < 0.001). The respiration rate did not significantly change over time (Additional file 1: Fig. S9, *p* = 0.16). In ARDS patients without cirrhosis, the tidal volume decreased over time (Additional file 1: Fig. S4, *p* < 0.001), the PEEP increased over time (Additional file 1: Fig. S5, *p* < 0.001), the plateau pressure decreased over time (Additional file 1: Fig. S6, *p* < 0.001), the driving pressure decreased over time (Additional file 1: Fig. S7, *p* < 0.001), the Crs increased over time (Additional file 1: Fig. S8, *p* = 0.04) and the respiration rate decreased over time (Additional file 1: Fig. S9, *p* < 0.001).

## Discussion

In this monocentric cohort of 863 consecutive ARDS patients from 2003 to 2021 who received protective lung ventilation, the 90 day mortality was of 43% (378/863). The mortality among ARDS patients with cirrhosis was 57% (90/157), compared to 41% for ARDS patients without cirrhosis (288/706). Cirrhosis was independently associated with mortality, after adjustment for age, non-hepatic SOFA and PaO2/FiO2 at day one (HR = 1.56, 95%CI [1.20–2.02]). However, the SOFA score and the non-hepatic SOFA score have increased in ARDS patients without cirrhosis, suggesting a greater initial severity of the patients. In the meantime, the severity of ARDS patients with cirrhosis has remained stable. Ventilatory parameters in ARDS patients with cirrhosis were comparable to ventilatory parameters in ARDS patients without cirrhosis.

To our knowledge, the present study reported the largest sample size of consecutive ARDS patients with cirrhosis (*n* = 157) over an 18-year period in a single medico-surgical ICU. Monchi and al. [5] reported a 96% mortality rate in a cohort of 27 ARDS patients with cirrhosis published in 1998 before the introduction of the protective lung ventilation [36]. Matuschak and al. [4] reported a 93% mortality rate in ARDS patients with cirrhosis awaiting liver transplantation. In 2016, Gacouin and al. [21] reported a 62% mortality rate in a cohort of 42 ARDS patients with cirrhosis, fairly similar to the 57% found in the present study.

The mortality was significantly different between ARDS patients with and without cirrhosis from day 7 and until day 90. The higher mortality in ARDS patients with cirrhosis found in this study might be explained either by the liver failure, by the inadequate ventilatory settings, or by the extrapulmonary organ failures. Acute on chronic liver failure (ACLF) is associated with higher mortality [37]. In our study, 62% of the ARDS patients with cirrhosis were classified grade 3 ACLF. Hence the hepatic disease evolution rather than the ARDS could explain the higher mortality in patients with cirrhosis. However, since ARDS patients with cirrhosis were excluded from 60% of the landmark randomized controlled trials on ARDS, their physiological specificities might need a tailoring of the ventilation settings in order to improve their outcomes [15]. Moreover, mechanical ventilation even without pulmonary failure has been shown to be associated with mortality in patients with cirrhosis, suggesting its deleterious effect on outcomes [38]. In ARDS patients with cirrhosis, the SOFA score and the non-hepatic SOFA score were higher than in ARDS patients without cirrhosis, although no significant difference was found in the PaO_2_/FiO_2_ at day one. In the same way, Gacouin and al. [21] found that the SOFA score without respiratory points was higher in ARDS patients with cirrhosis. Therefore, we could speculate that extrapulmonary organ failures play an important role in the outcome of ARDS patients with cirrhosis [37].

In ARDS patients without cirrhosis, the overall mortality in the present study of 43% is comparable with the existing literature on the main ARDS studies [2, 3, 15, 39–43] and with the Lung Safe study [1], in a range of 35 to 47%. There are discrepancies in the literature about the evolution over time of the mortality of ARDS patients. The papers that reported an improvement of ARDS outcomes over the last decade included mainly RCTs with selected patients [13]. Albeit, the papers which reported that the mortality remained stable over the last decades included non-interventional observational studies with consecutive non selected patients [14, 44, 45]. Unlike in ARDS, the prognosis of patients with cirrhosis admitted for septic shock has improved in the last decades [46].

In our study, 90% of the patients received a Vt < 8 ml/kg and less than 5% of the patients had a plateau pressure over 28 cmH2O, indicating that lung-protective ventilation was applied in our ICU during the study period. There was no difference in ventilation parameters between ARDS patients with and without cirrhosis. Patients with cirrhosis have specific conditions [47] (intraabdominal hypertension, ascites, pleural effusion), and their optimal ventilation settings could differ from that of the overall population. A specific assessment of the phenotype of ARDS in patients with cirrhosis could help to better manage those patients.

The main strength of the present study is the large sample of consecutive ARDS patients in a single center over an 18-year period (*n *= 863), which contains the largest sample of ARDS patients with cirrhosis (*n* = 157) ever published. Moreover, in this cohort, patients were managed with lung-protective ventilation. Finally, the results found in the multivariate Cox-proportional hazard model were confirmed in the exploratory multivariate model using logistic regression.

Our study has several limitations, starting with the monocentric design that hampers the generalization of the results. Second, although the data were prospectively recorded, the analysis is retrospective. It might lead to inhomogeneity of the population that could impact the evolution of the outcomes of ARDS patients with cirrhosis. We could not assess the proportion of ARDS caused by community-acquired pneumonia, although it might be a factor of poor prognosis in patients with cirrhosis [48]. We did not assess the CLIF-C ACLF and the CLIF-C AD scores, although we assessed the CLIF-C OF and ACLF grade. Last, we cannot rule out that ARDS patients with cirrhosis received less aggressive organ support than ARDS patients without cirrhosis. SOFA, non-hepatic SOFA and ARDS severity were greater in ARDS patients with cirrhosis. For this reason, we performed both the primary multivariate Cox-proportional hazard model and the exploratory multivariate logistic regression adjusting for non-hepatic SOFA and ARDS severity. Nitric oxide use was higher in ARDS patients without cirrhosis, even if it is not a first-line therapy in ARDS. Corticosteroids were administered much more frequently in ARDS patients with cirrhosis. Indeed, acute alcoholic hepatitis and auto-immune hepatitis were treated with corticosteroids regardless of the ARDS. Additionally, the prevalence of critical-illness-related corticoid insufficiency is very high in patients with cirrhosis, and those patients were treated with hydrocortisone. Causes of mortality and proportion of do-not-resuscitate orders were not recorded in this study. One could argue that do-not resuscitate orders might be more common in ARDS patients with cirrhosis. To partially overcome this bias, we chose to focus the study on ARDS patients receiving invasive mechanical ventilation, thus excluding patients with do-not-intubate orders. Moreover, MV-free days at day 28, the use of NMBA and prone positioning were not significantly different in both groups, and the ICU length of stay was similar in both groups.

## Conclusions

In this cohort of 863 ARDS patients from 2003 to 2021 who received protective lung ventilation, ARDS patients with cirrhosis had a significantly higher mortality than ARDS patients without cirrhosis. Although ARDS management improved over the last decades, the 90 day mortality remained high and stable over time for both ARDS patients with (57%) and without cirrhosis (41%). Nevertheless, the severity of patients without cirrhosis has increased over time, while the severity of patients with cirrhosis has remained stable. Improving the prognosis of ARDS patients with cirrhosis might require both tailoring ventilation settings and improving the management of other organ failures.

### Supplementary Information

**Additional file 1: Table S1.** Baseline comorbidities of 863 ARDS patients with cirrhosis. **Table S2.** Baseline characteristics of 157 ARDS patients with cirrhosis. **Table S3.** Missing values for studied outcomes. Table S4. Multivariate Cox-proportional hazard model. **Table S5.** Univariate analysis for 90 day mortality in ARDS patients with and without cirrhosis. **Table S6.** Multivariate model for 90 day mortality in ARDS patients. **Table S7.** Ventilatory parameters at day one of ARDS onset for ARDS patients with cirrhosis and ARDS patients without cirrhosis. **Figure S1.** Sensitivity analysis excluding 142 patients included in randomized trials: Cumulative 90 day mortality in 592 ARDS patients without cirrhosis and 129 ARDS patients with cirrhosis. **Figure S2.** Cumulative frequency graph of the compliance of the respiratory system in ARDS patients with cirrhosis and ARDS patients without cirrhosis. **Figure S3.** Cumulative frequency graph of the respiration rate in ARDS patients with cirrhosis and ARDS patients without cirrhosis. **Figure S4.** Time trends of the tidal volume over the study period ARDS patients with and without cirrhosis. **Figure S5.** Time trends of the positive end-expiratory pressure over the study period ARDS patients with and without cirrhosis. **Figure S6.** Time trends of the plateau pressure over the study period ARDS patients with and without cirrhosis. **Figure S7.** Time trends of the driving pressure over the study period ARDS patients with and without cirrhosis. **Figure S8.** Time trends of the compliance of the respiratory system over the study period ARDS patients with and without cirrhosis. **Figure S9.** Time trends of the respiratory rate of the respiratory system over the study period ARDS patients with and without cirrhosis.

## Data Availability

Research data and other material (e.g., study protocol and statistical analysis plan) will be made available to the scientific community, immediately on publication, with as few restrictions as possible. All requests should be submitted to the corresponding author who will review with the other investigators for consideration. A data use agreement will be required before the release of participant data and institutional review board approval as appropriate.

## Abbreviations

ARDS: Acute respiratory distress syndrome
ICU: Intensive care unit
PaO2/FiO2: PaO_2_/FiO_2_ ratio
SAPS II: Simplified Acute Physiology Score II
SOFA: Sequential Organ Failure Assessment
Vt: Tidal volume
PEEP: Positive end-expiratory pressure
Crs: Compliance of the respiratory system
MELD: Model for end-stage liver disease
PBW: Predicted body-weight
NMBA: Neuromuscular blocking agent
