# Supplementary material for: Outcomes and time trends of acute respiratory distress syndrome patients with and without liver cirrhosis: an observational cohort
Source: Ann Intensive Care. 2023 Sep 29;13:96. doi: 10.1186/s13613-023-01190-1 (PMC10541379; doi:10.1186/s13613-023-01190-1)

**ELECTRONIC SUPPLEMENTARY MATERIAL - SUMMARY**

**Table S1. Baseline comorbidities of 863 ARDS patients with cirrhosis**

**Table S2. Baseline characteristics of 157 ARDS patients with cirrhosis**

**Table S3. Missing values for studied outcomes**

**Table S4. Multivariate Cox-proportional hazard model**

**Table S5. Univariate analysis for 90-day mortality in ARDS patients with and without cirrhosis**

**Table S6. Multivariate model for 90-day mortality in ARDS patients**

**Table S7. Ventilatory parameters at day one of ARDS onset for ARDS patients with cirrhosis and ARDS patients without cirrhosis**

**Figure S1. Sensitivity analysis excluding 142 patients included in randomized trials: Cumulative 90-day mortality in 592 ARDS patients without cirrhosis and 129 ARDS patients with cirrhosis.**

**Figure S2. Cumulative frequency graph of the compliance of the respiratory system in ARDS patients with cirrhosis and ARDS patients without cirrhosis**

**Figure S3. Cumulative frequency graph of the respiration rate in ARDS patients with cirrhosis and ARDS patients without cirrhosis**

**Figure S4. Time trends of the tidal volume over the study period ARDS patients with and without cirrhosis**

**Figure S5. Time trends of the positive end-expiratory pressure over the study period ARDS patients with and without cirrhosis**

**Figure S6. Time trends of the plateau pressure over the study period ARDS patients with and without cirrhosis**

**Figure S7. Time trends of the driving pressure over the study period ARDS patients with and without cirrhosis**

**Figure S8. Time trends of the compliance of the respiratory system over the study period ARDS patients with and without cirrhosis**

**Figure S9. Time trends of the respiratory rate of the respiratory system over the study period ARDS patients with and without cirrhosis**

**Table S1. Baseline comorbidities in 863 ARDS patients with and without cirrhosis**

| **Characteristics** | **Total (%) (n=863)** | **Patients with cirrhosis (n=157)** | **Patients without cirrhosis (n=706)** | | **P value** |
| --- | --- | --- | --- | --- | --- |
|  |  |  | | | |
| **COPD** | 125 (14%) | 21 (13%) | | 104 (15%) | 0.66 |
| **Chronic heart disease** | 155 (18%) | 21 (13%) | | 134 (19%) | 0.12 |
| **Chronic kidney disease (GFR < 60 ml/min/1.73m²) *** | 65 (8%) | 13 (8%) | | 52 (7%) | 0.69 |
| **Solid tumor** | 207 (23%) | 32 (20%) | | 175 (24%) | 0.26 |
| **Hematological malignancy** | 40 (5%) | 3 (2%) | | 37 (5%) | 0.07 |
| **Diabetes** | 156 (18%) | 25 (16%) | | 131 (19%) | 0.51 |

COPD: Chronic Obstructive Pulmonary Disease. GFR: Glomerular filtration rate.

* At least Grade 3 chronic kidney disease

**Table S2. Baseline characteristics of 157 ARDS patients with cirrhosis**

| **Characteristic** | **Total (%) (n=157)** |
| --- | --- |
|  |  |
| **Child-Pugh score** |  |
| *A* | 19 (12%) |
| *B* | 48 (31%) |
| *C* | 90 (57%) |
| *Median [IQR]* | 10 [8-12] |
| **MELD, median [IQR]** | 22 [15-32] |
| **CLIF-C OF (±SD) * (1)** | 13 ± 5 |
| **ACLF grade ** (2)** |  |
| *No ACLF* | 9 (6%) |
| *ACLF Grade 1* | 16 (10%) |
| *ACLF Grade 2* | 34 (22%) |
| *ACLF Grade 3* | 97 (62%) |
| **Main cause of cirrhosis** |  |
| *Alcohol* | 94 (59%) |
| *Viral hepatitis* | 40 (26%) |
| *Non-alcoholic steatohepatitis* | 9 (6%) |
| *Autoimmune* | 7 (5%) |
| *Biliary* | 5 (3%) |
| *Hemochromatosis* | 2 (1%) |
| **Prothrombin rate (%), median [IQR]** | 42 [29-57] |
| **Bilirubin (µmol/L), median [IQR]** | 64 [28-148] |
| **Creatinine (µmol/L), median [IQR]** | 119 [75-198] |
| **Liver transplantation after diagnosis of ARDS** | 31 (19%) |

MELD: Model for End-Stage Liver Disease, CLIF: Chronic Liver Failure, OF: Organ Failure ARDS: Acute Respiratory Distress Syndrome. *Forty (5%) missing values. ** One (1%) missing value.

Additional references

1. Jalan R, Saliba F, Pavesi M, Amoros A, Moreau R, Ginès P, Levesque E, Durand F, Angeli P, Caraceni P, Hopf C, Alessandria C, Rodriguez E, Solis-Muñoz P, Laleman W, Trebicka J, Zeuzem S, Gustot T, Mookerjee R, Elkrief L, Soriano G, Cordoba J, Morando F, Gerbes A, Agarwal B, Samuel D, Bernardi M, Arroyo V; CANONIC study investigators of the EASL-CLIF Consortium. Development and validation of a prognostic score to predict mortality in patients with acute-on-chronic liver failure. J Hepatol. 2014;61:1038-1047.
2. Moreau R, Jalan R, Gines P, Pavesi M, Angeli P, Cordoba J, Durand F, Gustot T, Saliba F, Domenicali M, Gerbes A, Wendon J, Alessandria C, Laleman W, Zeuzem S, Trebicka J, Bernardi M, Arroyo V; CANONIC Study Investigators of the EASL–CLIF Consortium. Acute-on-chronic liver failure is a distinct syndrome that develops in patients with acute decompensation of cirrhosis. Gastroenterology. 2013;144:1426-1437, 1437.e1.

**Table S3. Missing values for studied outcomes**

| **Outcomes** | **Available data overall (%) (n=863)** | **Patients with cirrhosis (n=157)** | **Patients without cirrhosis (n=706)** | |
| --- | --- | --- | --- | --- |
|  |  |  | | |
| **90-day mortality** | 863 (100%) | 157 (100%) | | 706 (100%) |
| **SOFA** | 823 (95%) | 149 (95%) | | 674 (95%) |
| **Non-hepatic SOFA** | 823 (95%) | 149 (95%) | | 674 (95%) |
| **Age** | 863 (100%) | 157 (100%) | | 706 (100%) |
| **PaO2/FiO2 at day one** | 863 (100%) | 157 (100%) | | 706 (100%) |
| **Year of ICU admission** | 863 (100%) | 157 (100%) | | 706 (100%) |
| **Tidal volume** | 785 (91%) | 149 (94%) | | 636 (90%) |
| **PEEP** | 549 (64%) | 107 (68%) | | 442 (63%) |
| **Plateau Pressure** | 564 (65%) | 106 (68%) | | 458 (65%) |
| **Driving Pressure** | 517 (60%) | 111 (71%) | | 406 (58%) |
| **Compliance of the respiratory system** | 517 (60%) | 111 (71%) | | 406 (58%) |
| **Respiration Rate** | 767 (89%) | 144 (92%) | | 623 (88%) |

SOFA: Sequential Organ Failure Assessment, PaO2/FiO2: PaO2/FiO2 ratio, PEEP: Positive End-Expiratory Pressure

**Table S4. Multivariate Cox-proportional hazard model**

| **Characteristics** | **Hazard Ratio** | **95% Confidence Interval** | **p-value** | **Variance Inflation Factor** |
| --- | --- | --- | --- | --- |
|  |  |  | | |
| **Cirrhosis** | 1.56 | [1.20 – 2.02] | <0.001 | 1.09 |
| **Age (per year)** | 1.02 | [1.02 – 1.03] | <0.001 | 1.07 |
| **Non-hepatic SOFA (per point)** | 1.12 | [1.09 – 1.16] | <0.001 | 1.07 |
| **PaO2/FiO2 at day one (mmHg)** |  |  |  |  |
| *≥ 200* | - | - | - |  |
| *(100 ; 200]* | 1.49 | [1.15 – 1.92] | <0.01 | 1.52 |
| *(0 ; 100]* | 2.22 | [1.64 – 3.03] | <0.001 | 1.57 |

SOFA: Sequential Organ Failure Assessment, P/F: PaO2/FiO2 ratio.

**Table S5. Univariate analysis for 90-day mortality in ARDS patients with and without cirrhosis**

| **Characteristics** | **Patients with cirrhosis (n=157)** | | |  | **Patients without cirrhosis (n=706)** | | |  |
| --- | --- | --- | --- | --- | --- | --- | --- | --- |
|  | **Survivors at day 90 (n=67)** | **Non-survivors at day 90 (n=90)** | | **P value** | **Survivors at day 90 (n=418)** | | **Non-survivors at day 90 (n=288)** | **P value** |
| **Age (years), median [IQR]** | 56 [49-65] | 58 [50-64] | 0.23 | | | 60 [49-71] | 68 [60-75] | <0.001 |
| **Sex** |  |  | 0.24 | | |  |  | 0.93 |
| *Male* | 49 (73%) | 70 (78%) |  | | | 282 (67%) | 196 (68% |  |
| *Female* | 18 (27%) | 20 (22%) |  | | | 136 (33%) | 92 (32%) |  |
| **Non-hepatic SOFA, median [IQR]** | 8 [10-13] | 12 [10-14] | <0.001 | | | 8 [7-11] | 10 [8-13] | <0.001 |
| **Cause of ARDS** |  |  | <0.001 | | |  |  | <0.001 |
| *Medical* | 27 (40%) | 75 (83%) |  | | | 246 (59%) | 132 (46%) |  |
| *Postoperative* | 40 (60%) | 15 (17%) |  | | | 172 (41%) | 156 (54%) |  |
| **Origin of ARDS** |  |  | 0.14 | | |  |  | 0.40 |
| *Pulmonary* | 40 (60%) | 42 (47%) |  | | | 241 (58%) | 156 (54%) |  |
| *Extrapulmonary* | 27 (40%) | 48 (53%) |  | | | 177 (42%) | 132 (46%) |  |
| **Severity of ARDS** |  |  | 0.19 | | |  |  | 0.04 |
| *Mild* | 24 (36%) | 21 (23%) |  | | | 201 (48%) | 69 (24%) |  |
| *Moderate* | 33 (49%) | 50 (55%) |  | | | 196 (47%) | 146 (51%) |  |
| *Severe* | 10 (15%) | 19 (22%) |  | | | 21 (5%) | 73 (25%) |  |
| **Prone positioning** | 21 (31%) | 26 (29%) | 0.87 | | | 163 (39%) | 107 (37%) | 0.96 |
| **NMBA** | 28 (42%) | 58 (64%) | <0.01 | | | 237 (57%) | 170 (59%) | 0.59 |

IQR: interquartile range, SD: standard deviation, SOFA: Sequential Organ Failure Assessment, ARDS: Acute Respiratory Distress Syndrome, NMBA: Neuromuscular Blocking Agent.

**Table S6. Multivariate model for 90-day mortality in ARDS patients**

| **Variable** | **β parameter** ^1^ | **Odd ratio** | **95%CI** | ***P* value** |
| --- | --- | --- | --- | --- |
| **Intercept** | -3.72 |  |  | <0.001 |
| **Cirrhosis** | 0.58 | 1.78 | [1.15 – 2.75] | <0.01 |
| **Age (per year)** | 0.03 | 1.03 | [1.02 – 1.04] | <0.001 |
| **Non-hepatic SOFA (per point)** | 0.14 | 1.15 | [1.09 – 1.21] | <0.001 |
| **ARDS severity** |  |  |  |  |
| *Mild* | Ref | 1 |  |  |
| *Moderate* | 0.60 | 1.82 | [1.24 – 2.68] | <0.01 |
| *Severe* | 0.76 | 2.13 | [1.29 – 3.51] | <0.01 |
| **NMBA** | -0.06 | 0.94 | [0.90 – 0.99] | 0.02 |

Abbreviations: OR: Odd Ratio, 95%CI: Confidence Interval at 95%, ARDS: Acute Respiratory Distress Syndrome.

^1^ β parameters: coefficients from the logistic regression model.

**Table S7. Ventilatory parameters at day one of ARDS onset for 157 ARDS patients with cirrhosis and 706 ARDS patients without cirrhosis**

| **Parameters** | **Total (%) (n=863)** | **Patients with cirrhosis (n=157)** | **Patients without cirrhosis (n=706)** | | **P value** |
| --- | --- | --- | --- | --- | --- |
|  |  |  | | | |
| **Expiratory Vt (ml/kg of PBW), median [IQR] ^1^** | 6.28 [5.86-7.00] | 6.23 [5.86-6.95] | | 6.29 [5.88-7.00] | 0.94 |
| **PEEP (cmH2O), median [IQR] ²** | 12 [10-14] | 12 [10-15] | | 12 [10-14] | 0.84 |
| **Plateau pressure (cmH2O), median [IQR] ^3^** | 22 [19-25] | 22 [19-24] | | 22 [20-25] | 0.44 |
| **Driving pressure (cmH2O), median [IQR] ^4^** | 10 [8-12] | 10 [7-13] | | 10 [8-12] | 0.35 |
| **Compliance of the respiratory system (ml/cmH2O) ^4^** | 41 [32-53] | 40 [32-56] | | 41 [32-53] | 0.96 |
| **Respiratory rate, median [IQR] ^5^** | 24 [20-27] | 24 [22-27] | | 24 [20-27] | 0.40 |

IQR: interquartile range, PEEP: Positive End-Expiratory Pressure, Vt = Tidal Volume, PBW: Predicted Body Weight. ^1^ 78 (9%) missing values. ² 314 (36%) missing values. ^3^299 (35%) missing values. ^4^346 (40%) missing values. ^5^ 96 (11%) missing values.

**Figure S1. Sensitivity analysis excluding 142 patients included in randomized trials: Cumulative 90-day mortality in 592 ARDS patients without cirrhosis and 129 ARDS patients with cirrhosis.**

**
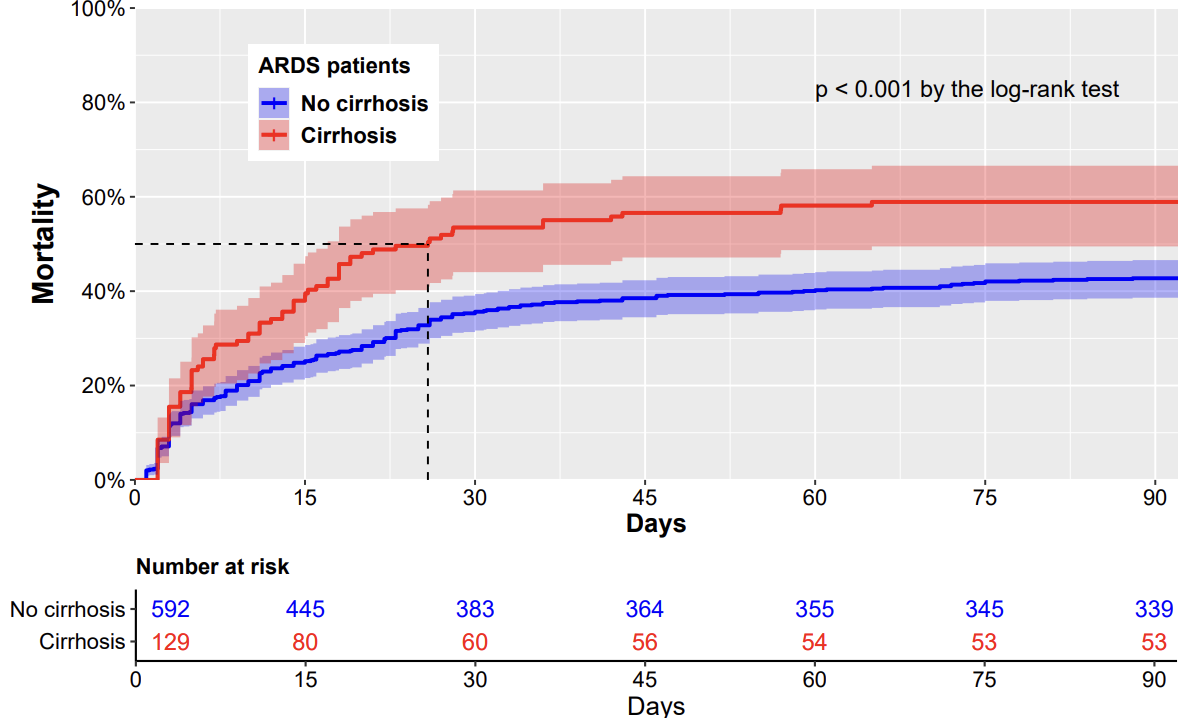
**

**Figure S2. Cumulative frequency graph of the compliance of the respiratory system in ARDS patients with cirrhosis and ARDS patients without cirrhosis**


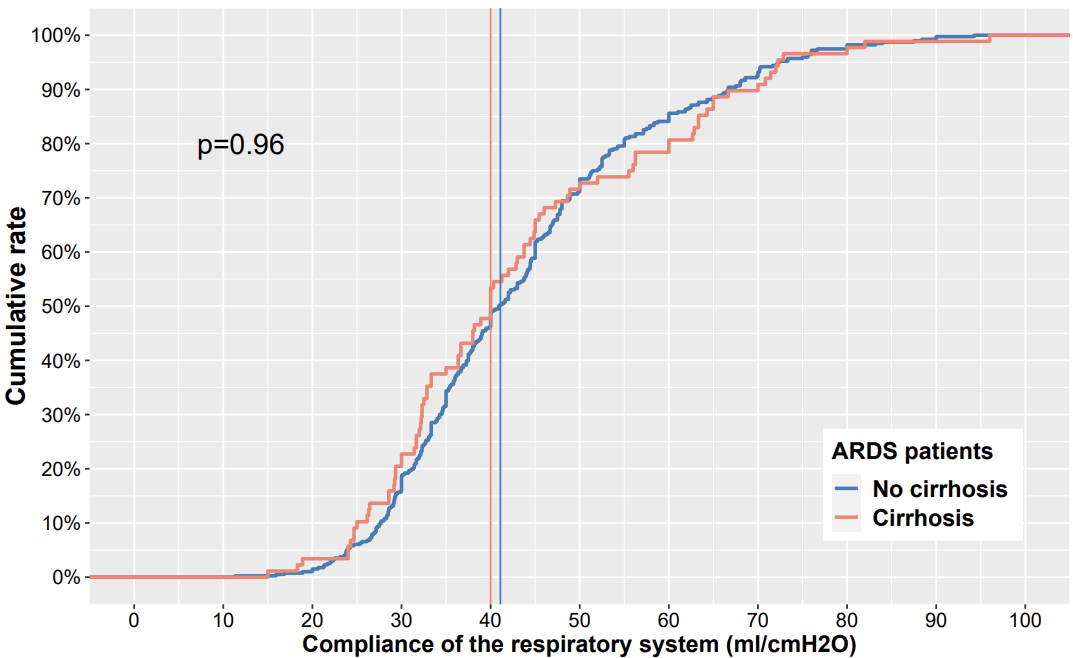


**Figure S3. Cumulative frequency graph of the respiration rate in ARDS patients with cirrhosis and ARDS patients without cirrhosis**


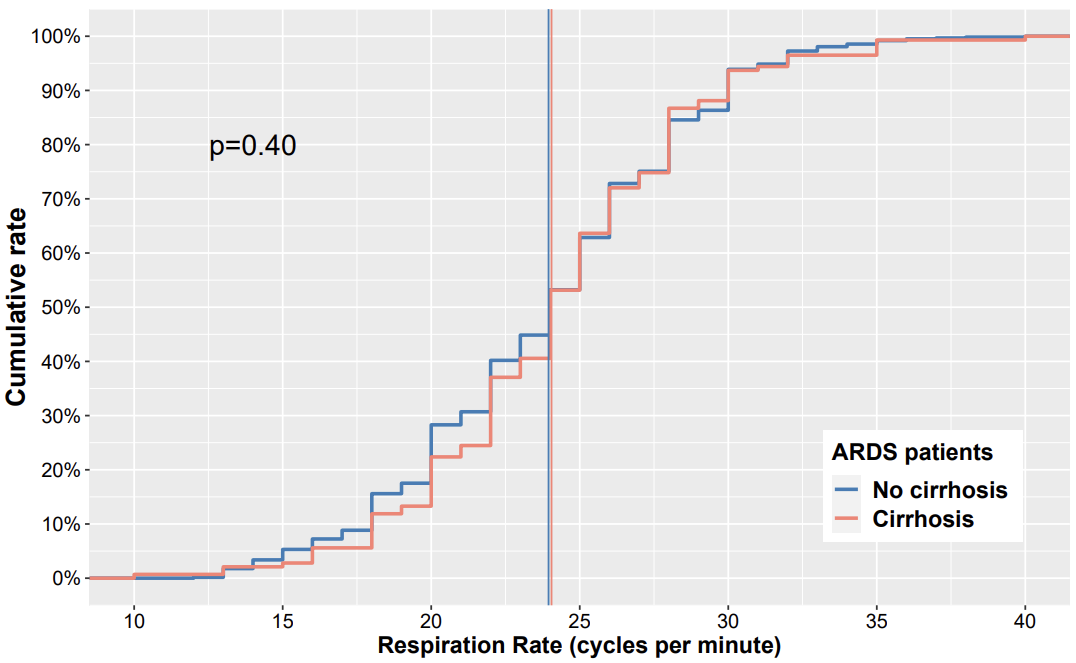


**Figure S4. Time trends of the tidal volume over the study period ARDS patients with and without cirrhosis**

1. **In ARDS patients with cirrhosis**
2. **In ARDS patients without cirrhosis**

PBW: Predicted Body Weight


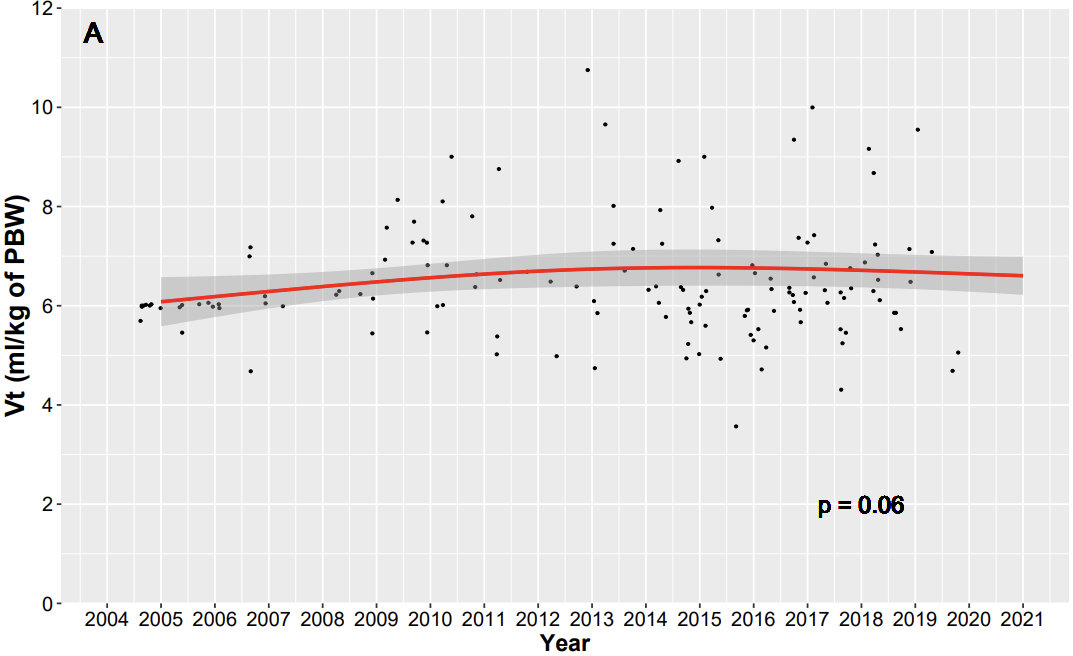


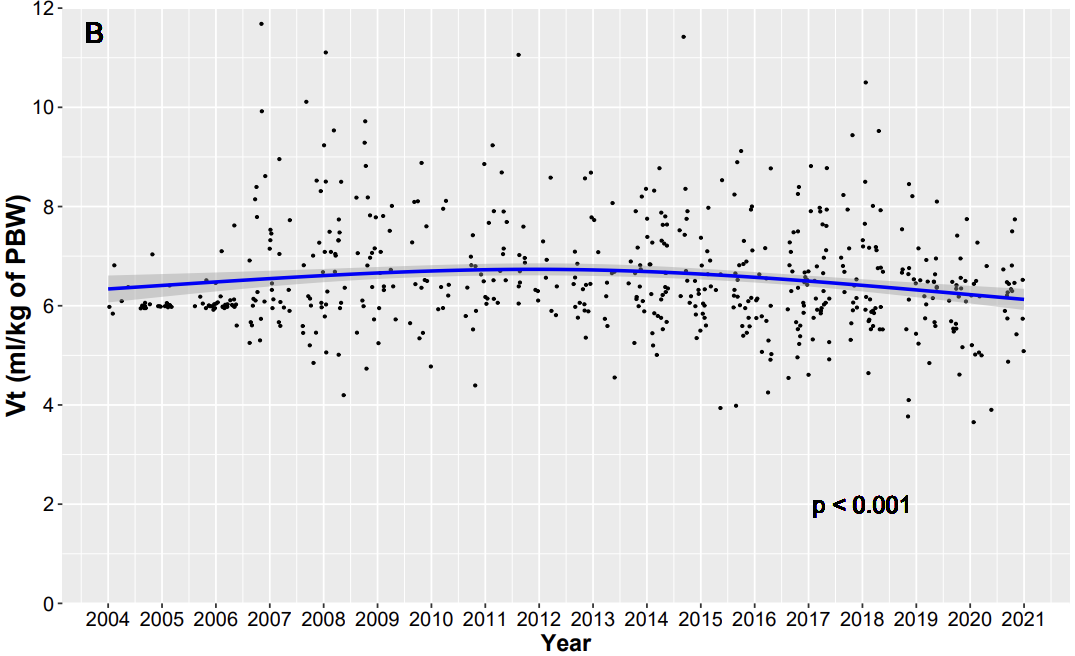


**Figure S5. Time trends of the positive end-expiratory pressure over the study period ARDS patients with and without cirrhosis**

1. **In ARDS patients with cirrhosis**
2. **In ARDS patients without cirrhosis**


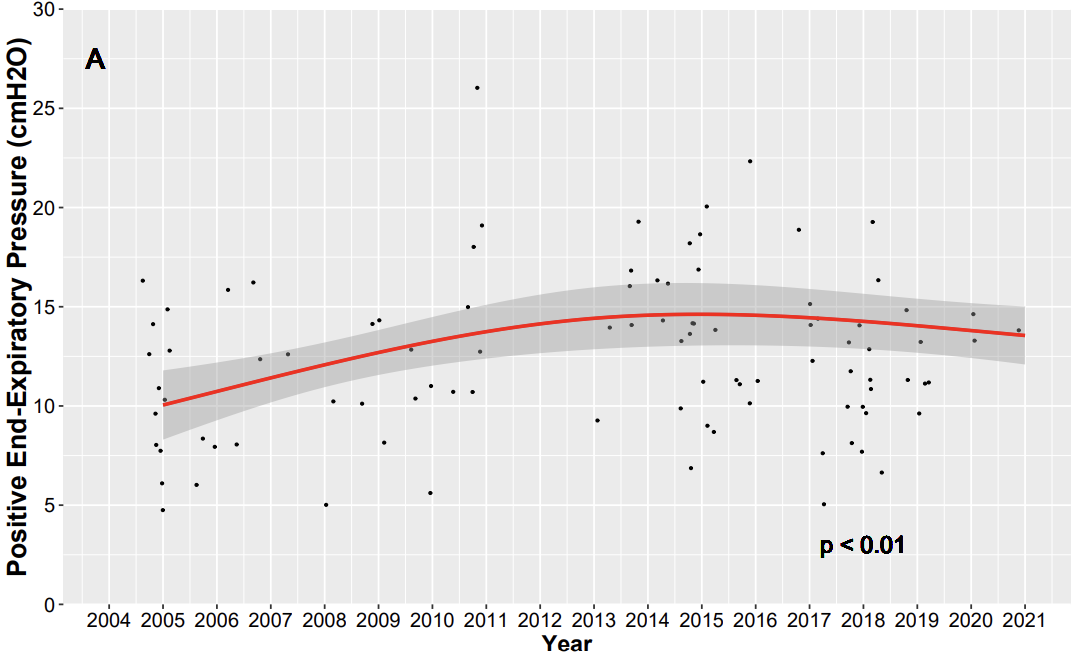


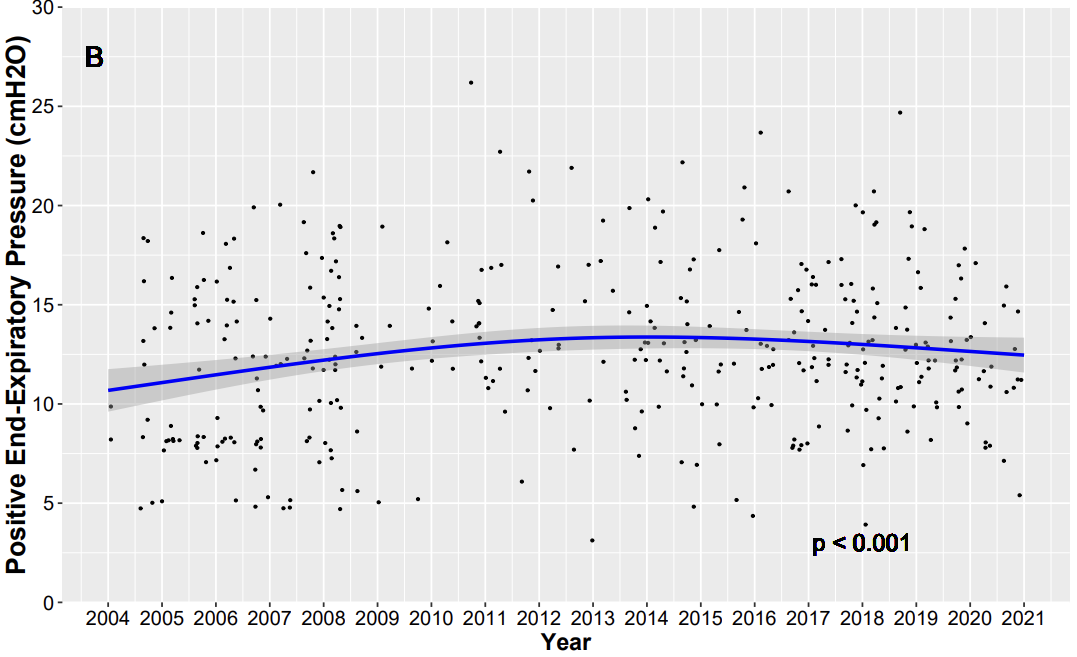


**Figure S6. Time trends of the plateau pressure over the study period ARDS patients with and without cirrhosis**

1. **In ARDS patients with cirrhosis**
2. **In ARDS patients without cirrhosis**


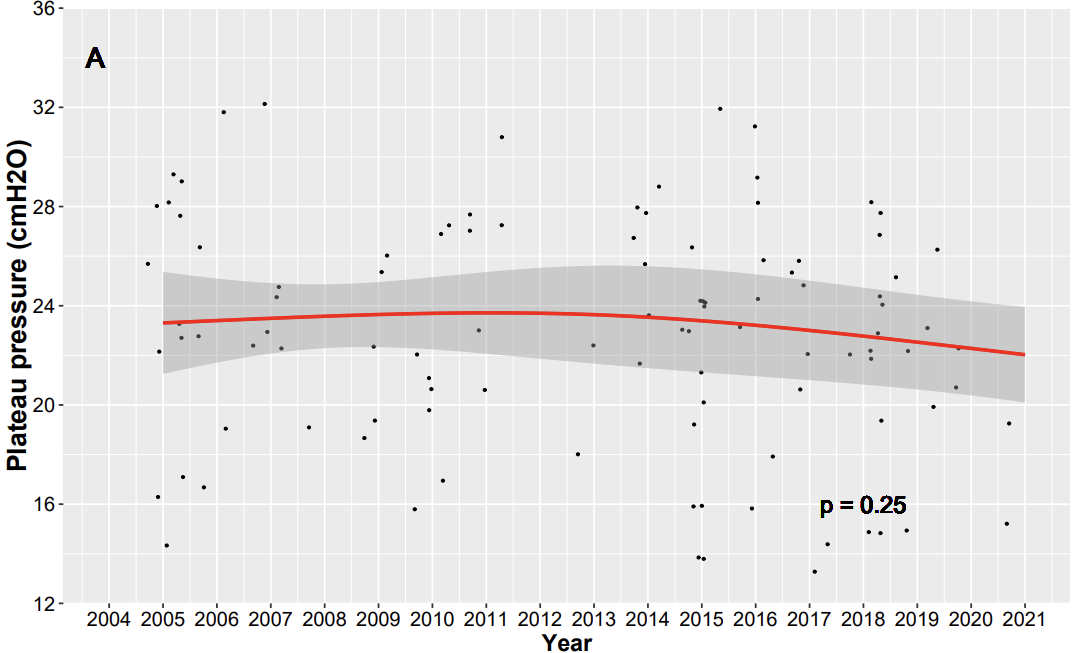


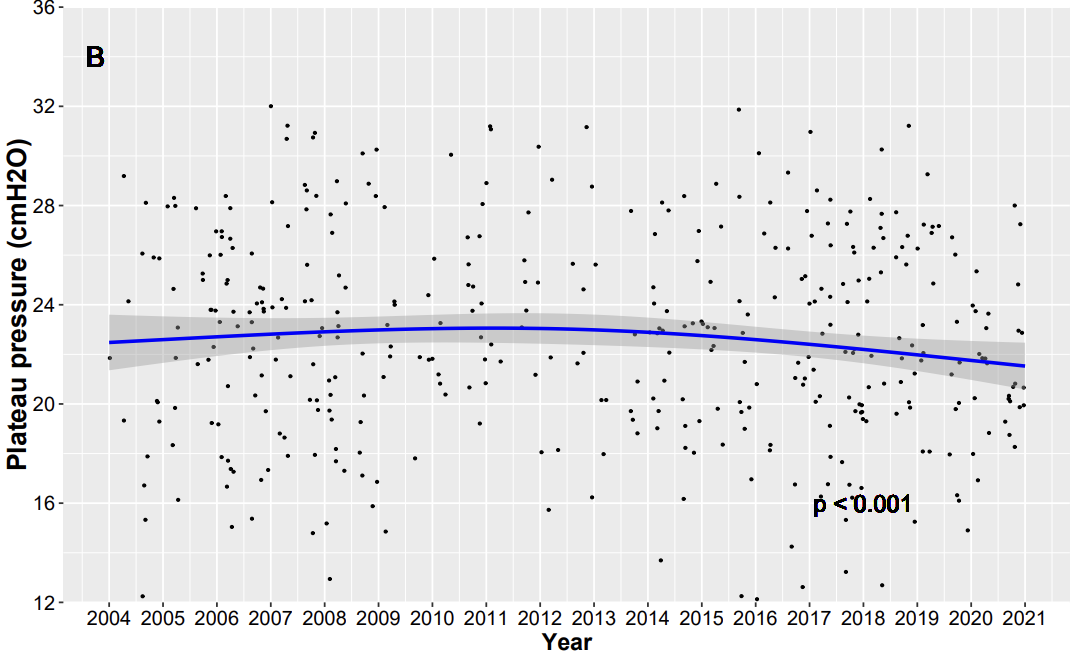


**Figure S7. Time trends of the driving pressure over the study period ARDS patients with and without cirrhosis**

1. **In ARDS patients with cirrhosis**
2. **In ARDS patients without cirrhosis**


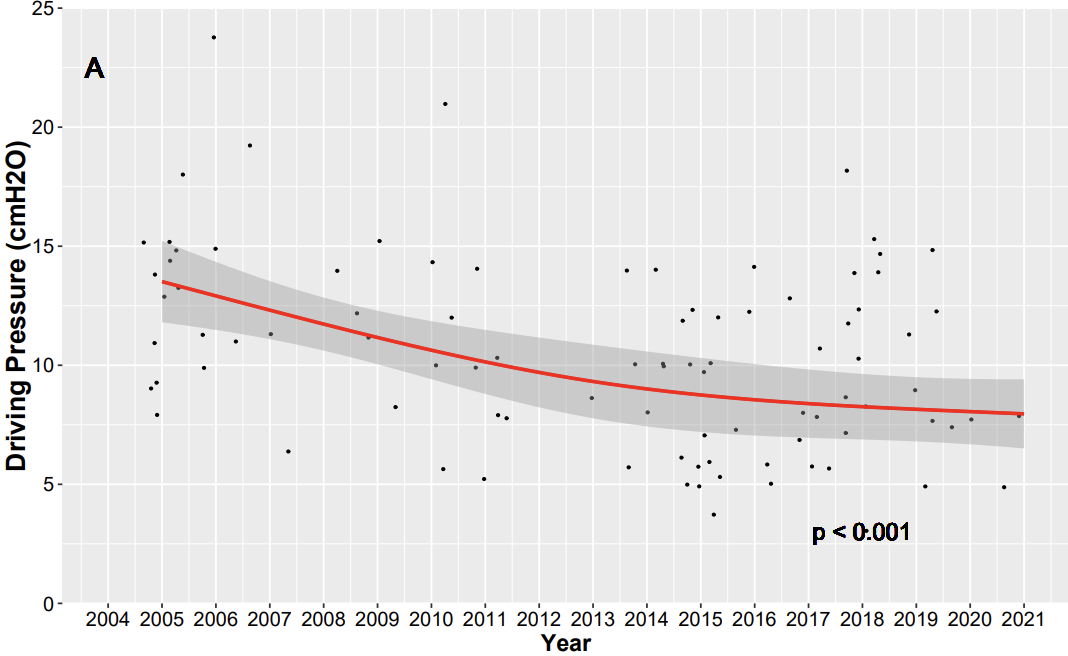


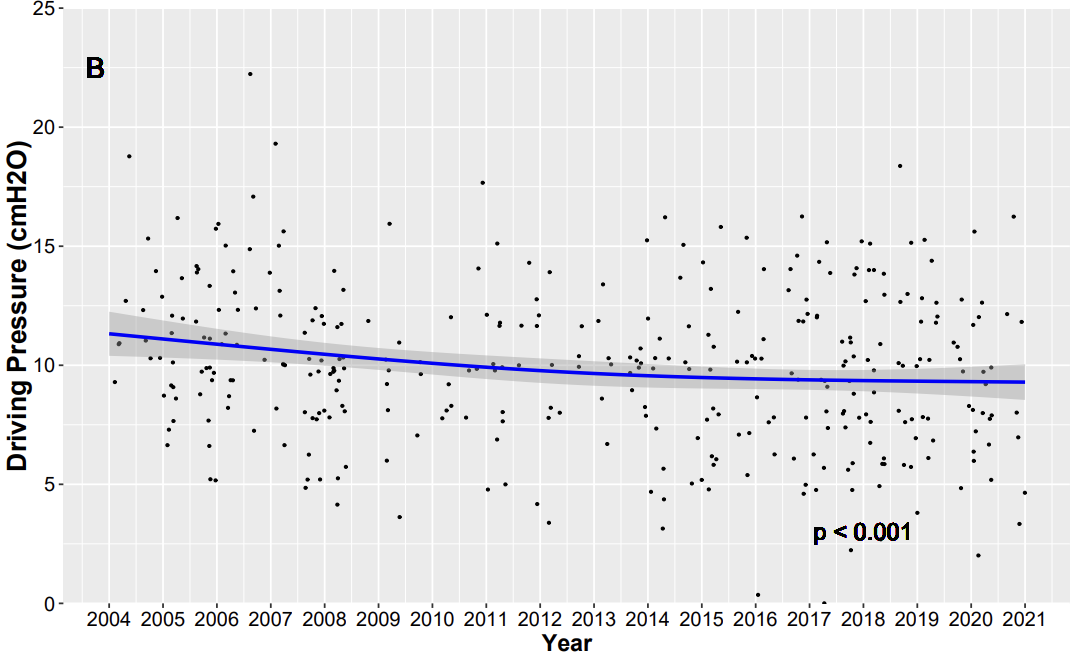


**Figure S8. Time trends of the compliance of the respiratory system over the study period ARDS patients with and without cirrhosis**

1. **In ARDS patients with cirrhosis**
2. **In ARDS patients without cirrhosis**


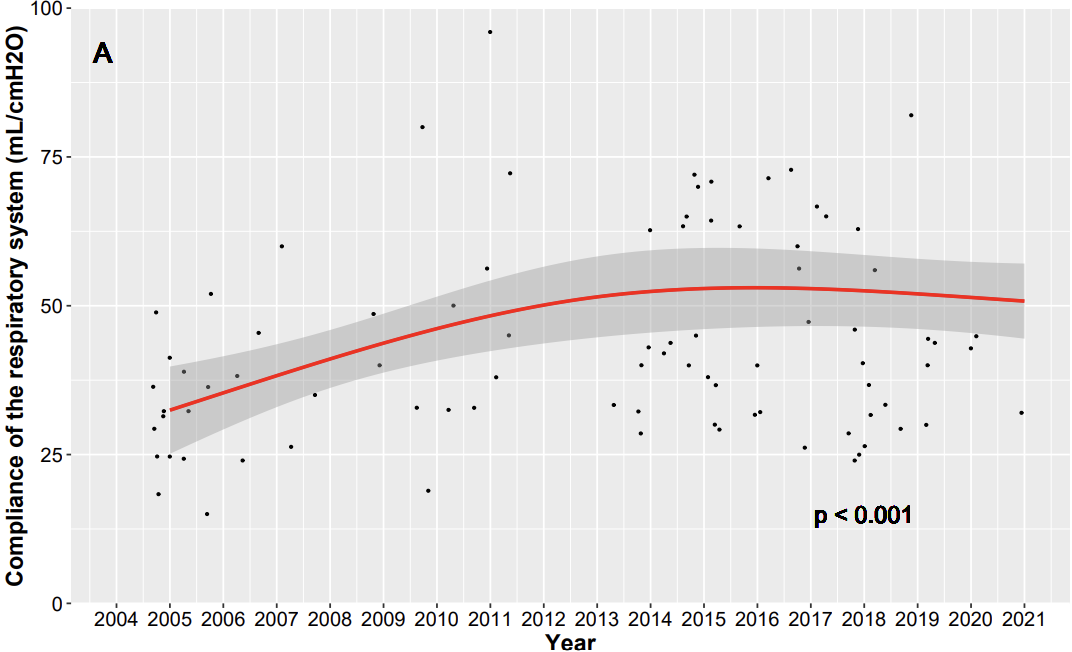


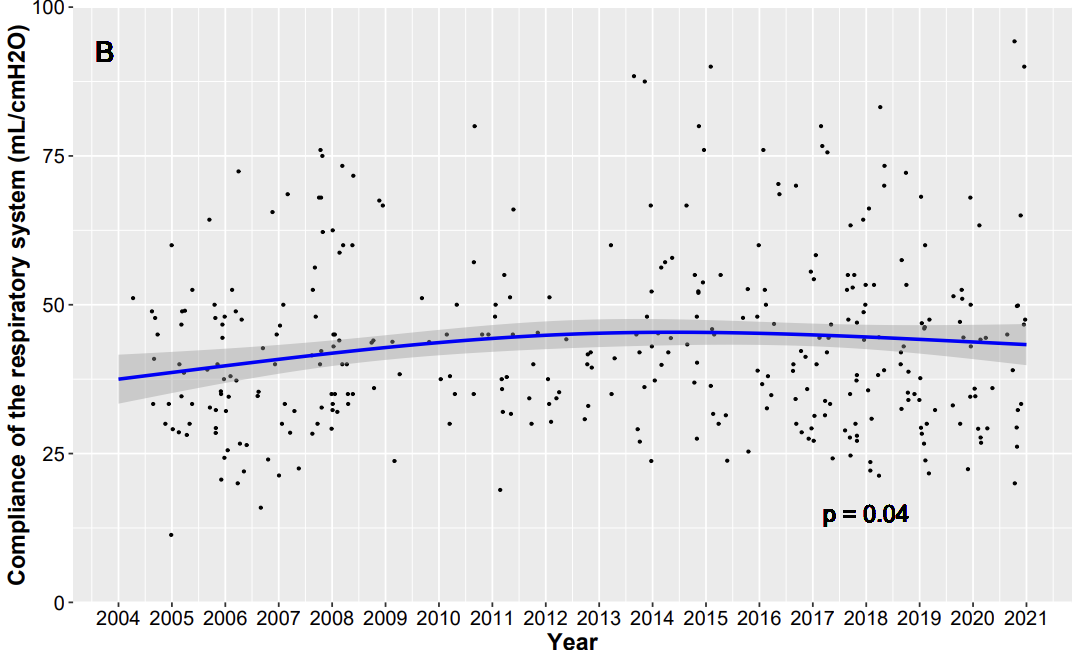


**Figure S9. Time trends of the respiratory rate over the study period ARDS patients with and without cirrhosis**

1. **In ARDS patients with cirrhosis**
2. **In ARDS patients without cirrhosis**


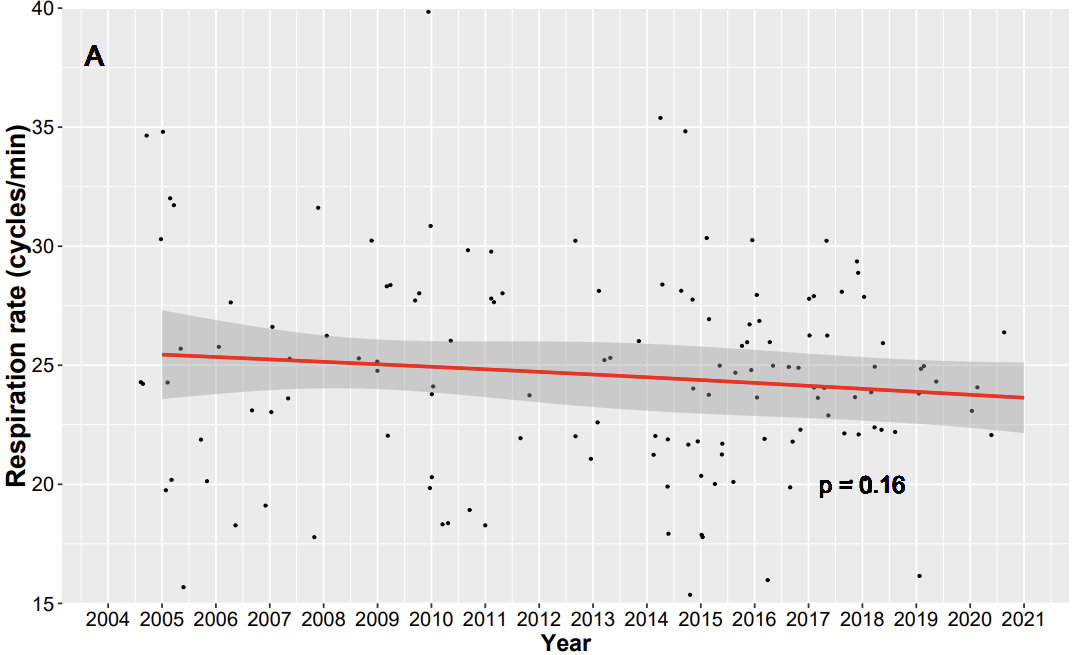


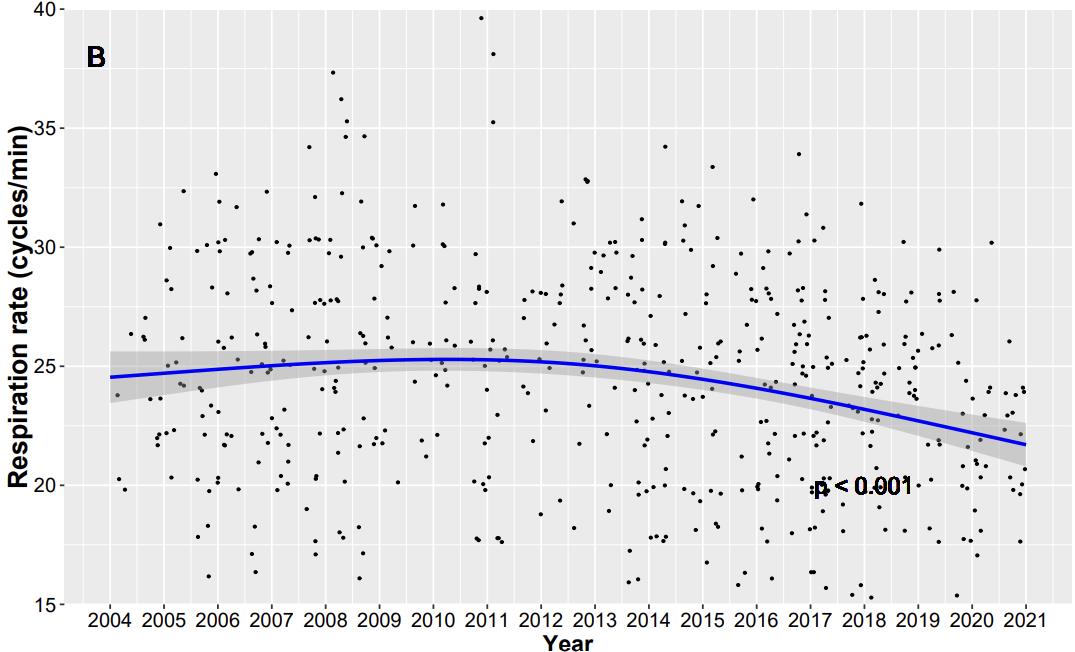

Supplement: Supplementary file 1 — Additional file 1: Table S1. Baseline comorbidities of 863 ARDS patients with cirrhosis. Table S2. Baseline characteristics of 157 ARDS patients with cirrhosis. Table S3. Missing values for studied outcomes. Table S4. Multivariate Cox-proportional hazard model. Table S5. Univariate analysis for 90 day mortality in ARDS patients with and without cirrhosis. Table S6. Multivariate model for 90 day mortality in ARDS patients. Table S7. Ventilatory parameters at day one of ARDS onset for ARDS patients with cirrhosis and ARDS patients without cirrhosis. Figure S1. Sensitivity analysis excluding 142 patients included in randomized trials: Cumulative 90 day mortality in 592 ARDS patients without cirrhosis and 129 ARDS patients with cirrhosis. Figure S2. Cumulative frequency graph of the compliance of the respiratory system in ARDS patients with cirrhosis and ARDS patients without cirrhosis. Figure S3. Cumulative frequency graph of the respiration rate in ARDS patients with cirrhosis and ARDS patients without cirrhosis. Figure S4. Time trends of the tidal volume over the study period ARDS patients with and without cirrhosis. Figure S5. Time trends of the positive end-expiratory pressure over the study period ARDS patients with and without cirrhosis. Figure S6. Time trends of the plateau pressure over the study period ARDS patients with and without cirrhosis. Figure S7. Time trends of the driving pressure over the study period ARDS patients with and without cirrhosis. Figure S8. Time trends of the compliance of the respiratory system over the study period ARDS patients with and without cirrhosis. Figure S9. Time trends of the respiratory rate of the respiratory system over the study period ARDS patients with and without cirrhosis. [file 13613_2023_1190_MOESM1_ESM.docx]
